# On cycles in the transcription network of Saccharomyces cerevisiae

**DOI:** 10.1186/1752-0509-2-12

**Published:** 2008-01-31

**Authors:** Jieun Jeong, Piotr Berman

**Affiliations:** 1 Department of Computer Science and Engineering, The Pennsylvania State University, University Park, PA 16802, USA

## Abstract

**Background:**

We investigate the cycles in the transcription network of *Saccharomyces cerevisiae*. Unlike a similar network of *Escherichia coli*, it contains many cycles. We characterize properties of these cycles and their place in the regulatory mechanism of the cell.

**Results:**

Almost all cycles in the transcription network of *Saccharomyces cerevisiae *are contained in a single *strongly connected component*, which we call LSCC (L for "largest"), except for a single cycle of two transcription factors. The fact that LSCC includes almost all cycles is well explained by the properties of a random graph with the same in- and out-degrees of the nodes.

Among different physiological conditions, cell cycle has the most significant relationship with LSCC, as the set of 64 transcription interactions that are active in all phases of the cell cycle has overlap of 27 with the interactions of LSCC (of which there are 49).

Conversely, if we remove the interactions that are active in all phases of the cell cycle (25% of interactions to transcription factors), the LSCC would have only three nodes and 5 edges, many fewer than expected. This subgraph of the transcription network consists mostly of interactions that are active only in the stress response subnetwork.

We also characterize the role of LSCC in the topology of the network. We show that LSCC can be used to define a natural hierarchy in the network and that in every physiological subnetwork LSCC plays a pivotal role.

**Conclusion:**

Apart from those well-defined conditions, the transcription network of *Saccharomyces cerevisiae *is devoid of cycles. It was observed that two conditions that were studied and that have no cycles of their own are *exogenous*: diauxic shift and DNA repair, while cell cycle and sporulation are *endogenous*. We claim that in a certain sense (slow recovery) stress response is *endogenous *as well.

## Background

Cycles have a central role in control of continuing processes (for an example, see Hartwell [1]). Therefore we expect the regulatory mechanism of a cell to have many cycles of interactions. Only some of these interactions have the form of a transcription factor (TF for short) regulating expression of a target gene. Our question is: given that there are cycles of transcription interactions, are they important in the regulation of life processes?

Graph properties of the regulatory networks have been reported in a number of papers. Shen-Orr *et al*. [2] analyzed the regulatory networks statistically and observed certain characteristic *motifs *that are more frequent than in the random model and which have functional significance (while other small subgraphs are significantly less frequent). Cycles, or feedback loops also may have some typical regulatory role, e.g. they may be related to multiple steady states [3-5].

Luscombe *et al*. [6] studied the dynamics of the regulatory network of *Saccharomyces cerevisiae *as it changes for multiple conditions and proposed a method for the statistical analysis of network dynamics. They have found large changes in the topology of the network and compared it with random graphs. We have found that the transcription network of *Saccharomyces cerevisiae *contains a single large strongly connected component (a union of overlapping cycles), which we call LSCC, and that the topology changes discussed by Luscombe *et al*. [6] are well reflected within LSCC, in spite of its small size.

**Figure 1:**
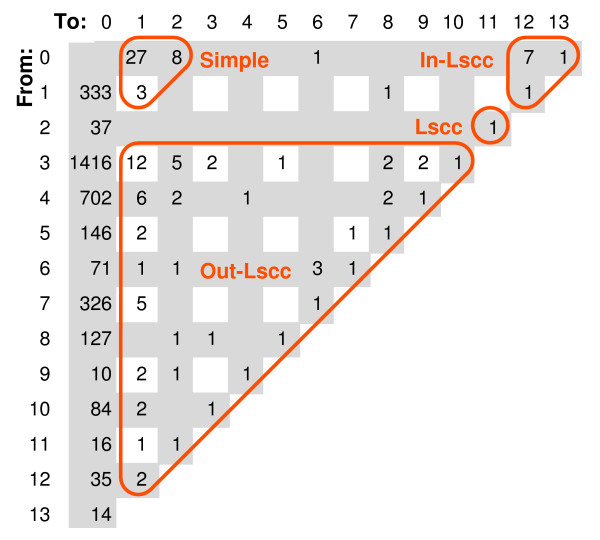
**Classifying TFs and TTs of Luscombe network by their positions on the longest paths, note that class INT is empty**. The paths are computed in the graph of scc's, in particular, we view LSCC as a single node. The entry in column *i *and row *j *shows the number of nodes with these properties: on the longest path through node *u *has *i *+ *j *edges and the longest path from *u ***to **another node (a TT) has *i *edges (consequently, the longest path **from **another node to *u *has *j *edges). Note that the only way a node may be on a path of length 3 is when it has an edge from the node that corresponds to LSCC.

**Figure 2:**
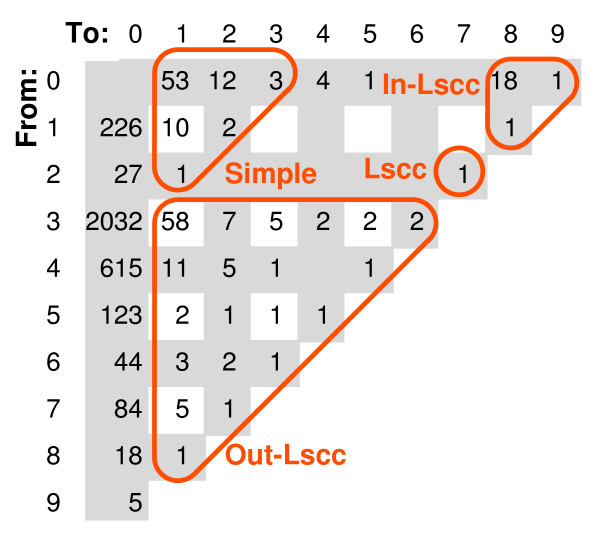
**Classifying TFs and TTs of Yu network by their positions on the longest paths, class INT is included in SIMPLE**. See explanations of Fig. 1.

Yu and Gerstein [7] have examined the structure of regulatory networks and showed that it exhibited a certain natural hierarchy. We propose another hierarchical partition of the network: above the LSCC, the LSCC, below the LSCC and "parallel" to the LSCC (see Fig. 1, 2) and we show that this partition is in some sense natural.

Comparisons of biological networks with random graphs were subject of methodological investigations of Barabasi and Albert [8] who proposed a *scale-free *model. This model is difficult to apply here. While the networks we investigated have the key property of scale-free networks, i.e. they have many nodes with degree much higher than the average, the distribution of the degrees is too irregular to match with a particular power law. In a scale free network the ratio #{nodes with degrees *k *to 2*k *- 1} to #{nodes with degrees 2*k *to 4*k *- 1} is convergent, but in our networks it varies widely for different k's (for recent study of scale-free nature on biological networks, see also [9,10]). Therefore Milo *et al*. [11] (see also Newman *et al*. [12]) proposed several methods of generating graphs that have the same in- and out-degrees as the reference network. We used their "matching algorithm" whenever possible, as well as faster and somewhat biased variants.

## Results and Discussion

In the data set of Luscombe *et al*. [6] we can see the LSCC with 25 TFs and one small strongly connected component with two TFs.

To see if the cycles of the LSCC are significant, we checked how the topological changes of the transcription network during various physiological conditions are reflected inside the LSCC, we checked several graph characteristics of the TFs in the LSCC, and we compared the characteristics of the LSCC to the cycles in random networks.

### General characterization of the cycles

#### Size of LSCC is relatively small

The cycles form two connected components, one "degenerate", consisting of 2 TFs, and one "large", consisting of 25 TFs.

The degenerate component consists of two TFs with indistinguishable interactions that have self-loops, thus they are TFs of themselves, and of each other. This may be a result of a relatively recent gene duplication. Thus we will ignore this cycle in our discussions.

The size of the largest cyclic component, 25, is rather small compared with random models (averages 42–43), with p-value ca. 0.025. The number of nodes in the remaining cycles, 2, is not very different from the average (0.8 to 1.3).

By the way of contrast, the transcription network of *Escherichia coli *is either devoid of cycles or it contains very few of them (depending on the data set, see Cosentino Lagomarsino *et al*. [13]).

#### LSCC connected very strongly to the cell cycle

The transcription network reported by Luscombe *et al*. [6] has 142 TFs and 7074 interactions, of which we disregard 21 "self-loop" interactions of the remainder 254 are TF to TF; we use ITF to denote the latter set (interactions to transcription factors). 25 TFs and 49 interactions form the LSCC. The subnetworks associated with the 5 stages of the cell cycle have 64 interactions in common (we name this set CCC, "common to cell cycle"), all of them directed to TFs (hence in ITF) and 27 of them are present in the LSCC. If even one of these two sets, LSCC or CCC, is random, the expected number of common elements would be smaller than 13 (49 × 64/254) and the probability of |LSCC ∩ CCC| ≥ 27 would be below 10^-6 ^(estimated by binomial formula). This shows that LSCC is very strongly related to the cell cycle.

#### Cycles of subnetworks other than cell cycle

Stress response is special in the sense that it has cycles of its own, all of which involve YAP6 that is not active in any other subnetwork. It seems that the cyclic interaction of this TF with two other TFs is a differentiating part of stress response condition from other exogenous conditions, diauxic shift and DNA damage. The latter have similar sets of active interactions in LSCC, but they lack 5 interactions involving YAP6.

One cycle consists of 3 interactions that are common to all conditions, REB1 → SIN3 → HSF1 → REB1. Note that HSF1 is a Heat Stress Factor, very important in the stress response, but also in "basal level sustained transcription" (see Mager and Ferreira [14]). One possible role of cycles in stress response is slowing down the recovery transition from the stress condition, so it can last several hours [14]. During the recovery, sporulation and cell cycle activities are suppressed. In this sense, stress response is partially *endogenous *to use the classification of Luscombe *et al*. [6] (they group Cell Cycle and Sporulation as endogenous and the other conditions as exogenous).

#### LSCC has an orderly layout

**Figure 3:**
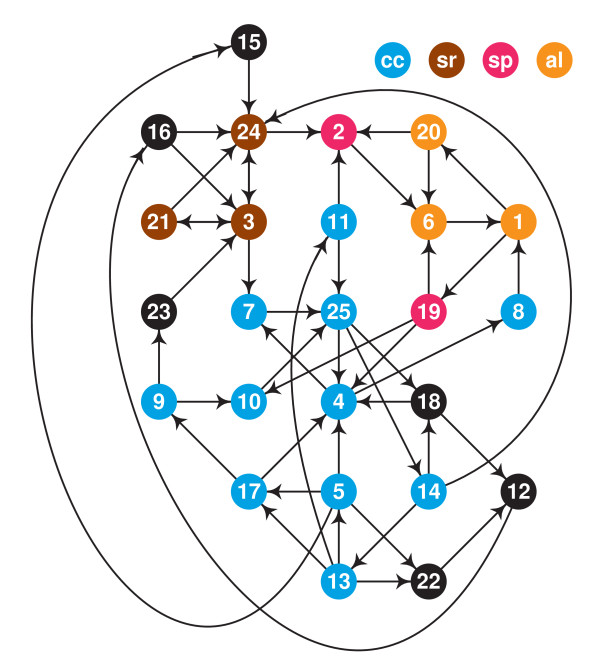
The diagram of LSCC, each node is TF in Table 6.

Fig. 3 shows the graph formed by the transcription factors and interactions of LSCC, with nodes placed on a square grid as to minimize the edge lengths.

In the diagram, **al **(apricot color) marks the nodes present in the cycles of all subnetworks. The cycles in the diauxic shift and DNA damage subnetworks contain only these nodes. (Note that an interaction of LSCC can be active in a subnetwork without belonging to a cycle in that subnetwork.)

The cycles in the sporulation subnetwork **sp **contain apricot and strawberry nodes.

The cycles in the cell cycle subnetwork **cc **contain apricot, strawberry and cerulean nodes.

The cycles in the stress response subnetwork **sr **contain apricot and sienna nodes.

Nodes that are not included in the cycles of any subnetwork are black.

We managed to find an orderly layout for LSCC, in which few edges are long while nodes with the same color are grouped together.

#### LSCC has small feedback vertex set

Another property of LSCC is that it has a small and unique minimum *feedback vertex set*, a set of nodes whose removal destroys all cycles.

**Figure 4:**
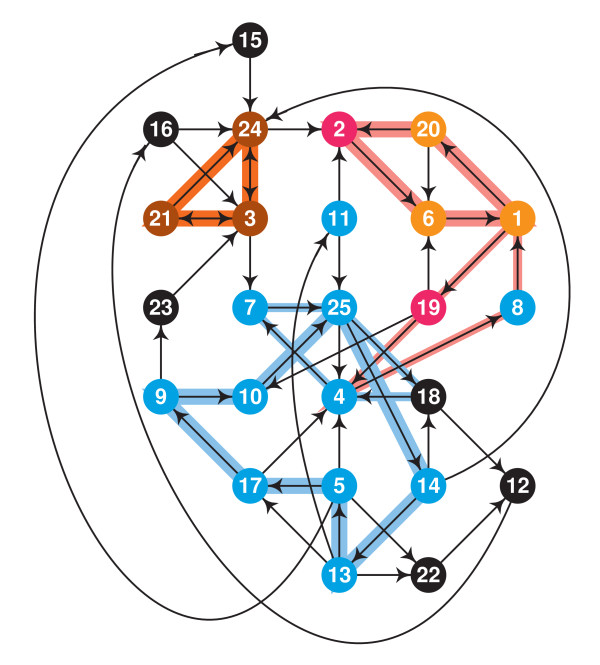
**The smallest feedback vertex set of LSCC and the subdivision of LSCC**. At least three feedback vertices are needed because there exists three vertex-disjoint cycles – indicated by wide color strips. If a single vertex selection on an indicated cycle suffices for the feedback vertex set then it must intersect every cycle that is vertex disjoint with the other indicated cycles; cycles indicated with thin color strips show that such selections are unique. A pictorial proof that {1, 3, 25} is the unique minimum feedback vertex set.

The fact that there exists a unique minimum feedback vertex set with three nodes (vertices) can be clearly seen in Fig. 4. Let us call this set *F *= {1, 3, 25}.

We can use *F *to distinguish three natural cyclic units within LSCC, *S*_*b *_for each *b *∈ *F*. We can think that *b *is the "boss" of *S*_*b*_. We define *S*_*b *_as the union of all simple cycles that go through *b *but not through *F *- {*b*}. Only one node can have two bosses: {4} = *S*_1 _∩ *S*_25_. Because there is only one path from 1 to 4 and three disjoint paths from 25 to 4, we remove 4 from *S*_1 _to make our units disjoint. The three sets coincide well with functional categories: *S*_3 _= {3, 21, 24} are the nodes on cycles of LSCC_**sr**_, *S*_1 _are the nodes on cycles of LSCC_**sp**_, and *S*_25 _are the nodes on cycles of LSCC_**cc **_minus *S*_1 _(observe that *S*1 is contained in LSCC_**cc**_). (Actually, *S*_25 _has 11 nodes and it has one node that is not in LSCC_**cc**_, 18, and one node of the cell cycle network is missed, 8.)

**Figure 5:**
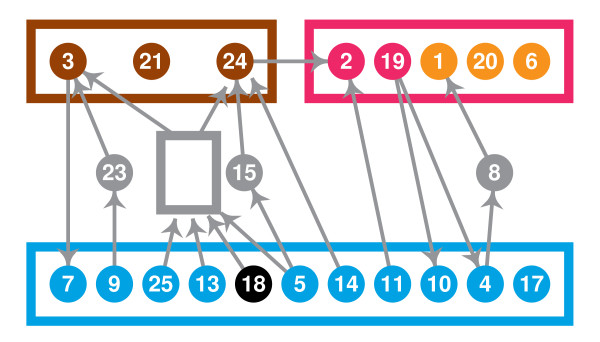
Three cyclic units of LSCC with connections.

Thus the cyclic subnework has three cyclic parts, plus two acyclic parts: 5 nodes on paths from *S*_25 _to *S*_3_, and 1 node on a path from *S*_25 _to *S*_1_. We show this schematically in Fig. 5.

#### Differences and similarities of subnetworks are reflected in LSCC

For subnetwork *A *we define LSCC_*A *_as the set of interactions of *A *that are also in LSCC; to measure the difference between two sets we use |*A *⊕ *B*|, the number of elements that are in one of the sets *A *and *B *but not in both.

**Figure 6:**
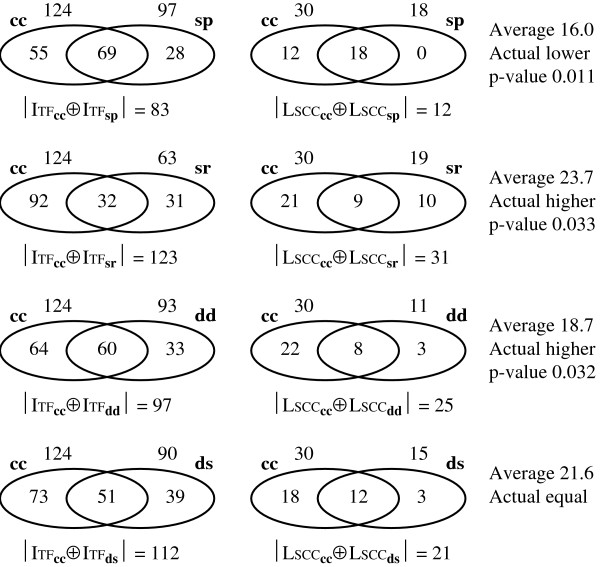
Intersections and symmetric differences of functional subnetworks inside ITF and LSCC.

One way that shows the importance of LSCC to regulatory mechanism is that the differences and similarities between physiological conditions tend to be "exaggerated" when we use LSCC as the "window". When we compare a symmetric difference of |ITF_**x **_⊕ ITF_**y**_| with |LSCC_**x **_⊕ LSCC_**y**_|, the size of the latter should be, on the average, 49*/*254 of the former. These comparisons are in Figure 6. In general, **sp **is very related to **cc**, and the difference inside LSCC is smaller than expected, while **dd**, **ds **and **sr **are unrelated, and the differences in LSCC are larger than expected, especially in the case of **sr**, the stress response.

### Statistic profile of the TFs from the LSCC for three different original networks

We tested properties of LSCC in randomly generated networks. We also tabulated results of random tests based on two larger data sets. In our tables, we refer to the networks using names of the first authors of the paper in which they were published [6,7,15], hence we call them Luscombe, Yu and Balaji.

In our random networks we kept all original connections from TFs to Terminal Targets (i.e. regulated genes which are not TFs themselves. Later we refer to them with abbreviation TTs). The remaining connections were "rewired" at random, using three criteria, R, F and B. Criterion R was a uniformly random permutation of the edge ends, conditional on obtaining a "correct network" – no self-loops or duplicated edges. Criterion F was creating a bias in the selection of the permutation so the resulting number of feed-forward loops was close to the actual value in the original network. Criterion B was similar, but with bi-fans rather than feed-forward loops.

When we refer to our computed average value we used form *x *(*y, z*) to denote "average obtained using criterion R (F, B)".

#### Average size and out-degree

The size of LSCC is quite a bit smaller than the average, 25 versus 42 (41, 43), with p-value of 0.025 (0.04, 0.02), and the situation is similar for Yu and Balaji. (The sizes of LSCC, as well as the classes defined in the next section in terms of LSCC, are in Tables 1, 2, 3.)

The average number of targets for the TFs of the LSCC is much higher than the average for all TFs, 128 versus 50. This discrepancy is somewhat smaller when we make such a comparison in a random model, 97 (96, 100) versus 50. Because cycles are sets of edges, it is very clear that a node with large out-degree has more chances to belong to a cycle, or a union of overlapping cycles that is LSCC. For example, in the actual network, almost half members of LSCC (12 of 25) belong to the top 20 TFs if we rank them by the number of the targets.

**Table 1:** Average sizes of classes compared with random model R

	IN-LSCC	LSCC	OUT-LSCC	SIMPLE	SSCC	INT	EXCP
	Luscombe						
actual	9	25	68	38	2	0	2
average	17.1	42.3	43.2	33.8	1.00	2.8	2.8
p-value	0.02	0.025	0.001	0.062	0.6	0.097	0.58
	Yu						
actual	20	63	114	77	5	6	5
average	32.5	69.5	102.8	69.6	0.44	6.3	4.2
p-value	0.001	0.002	0.020	0.22	0.01	0.32	0.34
	Balaji						
actual	21	60	58	14	0	3	1
average	20.9	74.4	45.6	14.3	0.2	1.2	0.5
p-value	0.53	0.002	0.002	0.57	0.92	0.14	0.35

We use Sscc to denote small cyclic scc's. The TFs that are neither in LSCC nor in its in- or out-components are classified according to MPL, the maximal path length for a path that includes a given TF; when MPL is 1 or 2, TF is in SIMPLE, if MPL is more than 3, TF is in EXCP, and if MPL is 3, we could make either decision, so here we inluded intermediate class INT.

Random graphs were produced to get a uniform distribution among graphs in which TFs have the same in-and out- degrees as in the original network, without changing the TF-TT connections.

**Table 2:** Average sizes of classes compared with random model F

	IN-LSCC	LSCC	OUT-LSCC	SIMPLE	SSCC	INT	EXCP
	Luscombe						
actual	9	25	68	38	2	0	2
average	16.2	40.7	45.0	34.1	1.25	2.9	3.15
p-value	0.043	0.041	0.004	0.081	0.30	0.086	0.555
	Yu						
actual	20	63	114	77	5	6	5
average	29.0	66.0	107.0	71.0	0.7	6.9	4.9
p-value	0.032	0.35	0.19	0.081	0.022	0.48	0.48
	Balaji						
actual	21	60	58	14	0	3	1
average	19.8	72.1	47.7	14.8	0.2	1.6	0.8
p-value	0.39	0.006	0.011	0.45	0.99	0.23	0.46

See explanations for Table 1. Generation of random graphs was altered to achieve the same number of feedforward loops as in the original network.

**Table 3:** Average sizes of classes compared with random model B

	IN-LSCC	LSCC	OUT-LSCC	SIMPLE	SSCC	INT	EXCP
	Luscombe						
actual	9	25	68	38	2	0	2
average	17.5	43.0	42.5	33.4	0.85	2.7	2.7
p-value	0.018	0.017	0.001	0.05	0.26	0.105	0.57
	Yu						
actual	20	63	114	77	5	6	5
average	33.7	67.5	99.7	71.8	0.43	7.4	4.9
p-value	0.002	0.26	0.025	0.12	0.013	0.4	0.47
	Balaji						
actual	21	60	58	14	0	3	1
average	22.6	74.2	43.8	14.4	0.2	1.4	0.7
p-value	0.39	0.003	0.000	0.56	0.91	0.18	0.46

See explanations for Table 1. Generation of random graphs was altered to achieve the same number of bi-fan motifs (a pair of TFs regulating the same pair of targets) as in the original network.

The lower average out-degree of the LSCC in random models is perhaps a simple consequence of the fact that they have, on the average, much larger LSCC, so the TFs from the top 20 TFs do not dominate the average as much as in the smaller LSCC of the actual network. Detailed comparisons of average out-degrees can be found in Table 4.

**Table 4:** Average out-degrees compared with three random models

	in LSCC						among all TFs	
	
	OUT			OUTF			OUT	OUTF
model	R	F	B	R	F	B		
					Luscombe			
actual		128.08			4.92		49.67	1.79
average	97.68	100.67	96.55	3.88	3.97	3.85	-	-
p-value	0.003	0.013	0.001	0.003	0.009	0.001	-	-
					Yu			
actual		85.54			6.35		29.27	2.01
average	75.08	79.26	75.61	5.68	5.96	5.71	-	-
p-value	0.030	0.148	0.032	0.037	0.169	0.038	-	-
					Balaji			
actual		146.78			4.87		81.99	3.12
average	131.61	134.80	130.69	5.31	5.43	5.26	-	-
p-value	0.002	0.015	0.002	0.005	0.031	0.009	-	-

In this table, we have separate columns for results of random tests according to model R, F and B respectively. Columns OUT give the average number of targets, and columns OUTF give the average number of targets that are TFs. Note that in random networks the number of targets is the same for each TFs, so the average values for all TFs are always the same.

#### Position of LSCC in the hierarchy

Only 9 TFs belong to the in-component of the LSCC (denoted In-LSCC) in the sense that there are paths from these TFs to the LSCC; of these 9 paths 8 are single edges and one consists of two edges. If we consider that path to be exception, collectively the LSCC has unambiguous hierarchical position 2nd from the top. In a random network, on the average we have 17 (16, 17.5) TFs in In-LSCC. In this sense, the LSCC is higher in the hierarchy than the average in the random models.

Almost all paths with more than 2 edges are related to the LSCC in the following sense: either they include a TF from the LSCC, or form the final part of a path that starts in the LSCC. Two TFs form an exception to that rule, namely they can start a path with more than 2 edges that is not such a final part.

After collapsing scc's to single nodes we measured for each TF the maximum path length (for paths to which it belongs), and we call it MPL. For 38 TFs the value of MPL is at most 2, and they form a rather separate part of the transcription network which we call SIMPLE. 104 TFs have MPL of at least 3. Maximum of MPL is 13, more than the average in random networks that is 8.3 (8.4, 8.5). (The maximum length of a simple path is perhaps a better measure, but it requires a much more complex program to compute it. It is closely related to the feedback vertex set problem.)

Yu and Gerstein [7] propose a partition of networks according to the length of shortest paths to those TFs that have only TTs as their targets. This definition would not work with the length of the shortest paths to TTs: this length is 1 for all TFs but ten, and for that ten, it is 2, so the hierarchy would be trivial. Because LSCC has such a special and statistically significant position in the network, we propose to partition TFs by their relation to LSCC, as it is indicated in Fig. 1. In particular, TFs with a path to or from LSCC are partitioned into hierarchy IN-LSCC, LSCC and the out-component of LSCC (denoted OUT-LSCC), while the remaining TFs are classified according to MPL; if MPL is at most 2, they are in SIMPLE, if it is more than 3, they are in EXCP, and if it is equal 3, we place them in the intermediate class INT (which is empty in Luscombe data set).

We performed our study using the data of Luscombe *et al*. [6] because we wanted to compare the cycles with physiological subnetworks described in their paper. Nevertheless, we compared our definition of a hierarchy with that of Yu and Gerstein [7], who performed their investigation in a larger transcription network.

When we apply our program to the latter network, the proportions between the class sizes remain similar (here we included INT in SIMPLE): IN-LSCC (20), LSCC (63), OUT-LSCC (114), SIMPLE (83) and EXCP (5). Tables 1, 2, 3 show detailed comparison of class sizes.

We performed two tests applied by Yu and Gerstein to their classes (see Fig. 2 for the partition of Yu network into classes).

When we checked the percentage of essential genes in our classes, we got 15% in IN-LSCC and LSCC, 13% in OUT-LSCC and 12% in SIMPLE, a more uniform distribution than among classes of Yu and Gerstein. A more striking difference exists when we check the percentage of cancer related genes: 10% in IN-LSCC, 9.5% in LSCC, 3.5% in SIMPLE and 2.6% in OUT-LSCC.

The division we propose is closely related to the notion proposed by Yu and Gerstein: a division of transcription control mechanisms into *reflex *processes and *cogitation *processes. SIMPLE clearly corresponds to reflex processes. In a cogitation process, one that involves a long path of interactions, we can partition the process into beginning, middle and the ending part. As the various paths have very different lengths, identifying LSCC as the middle is both "objective" and independent from the path length, and in the same time quite arbitrary. However, we show in the next subsection that LSCC has a "switchboard" property even in the physiological conditions in which paths do not form cycles, and we just have seen that the percentage of cancer related genes sharply drops as we move from the middle to the final part of the long paths.

### Topological changes inside LSCC

**Figure 7:**
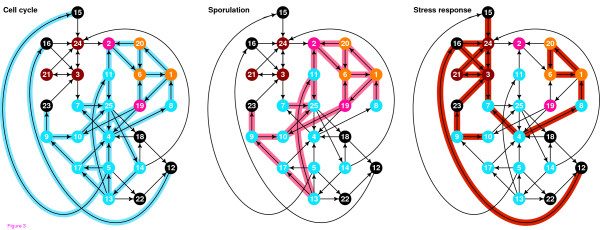
**Parts of LSCC that are active during endogenous condition (or, conditions with larger number of active cycles)**. Cell cycle: Interaction between 5 and 15 appears to repress stress response. Sporulation: Most of the cell cycle interactions are present, but the cycle interactions leaving node 25 are not. Replication of DNA is an activity shared with the cell cycle. Stress response: When we compare the part of LSCC that is active during stress response with parts of LSCC that are active during cell cycle and sporulation, we note that in the latter cases the stress response cycle is totally inactive, but it is partially active during the diauxic shift and DNA damage, which are related to stress (damage – obvious, diauxic shift – the shift toward less favored nutrition source). Center of the cell cycle is activated during stress response, which can be part of a repression mechanism.

**Figure 8:**
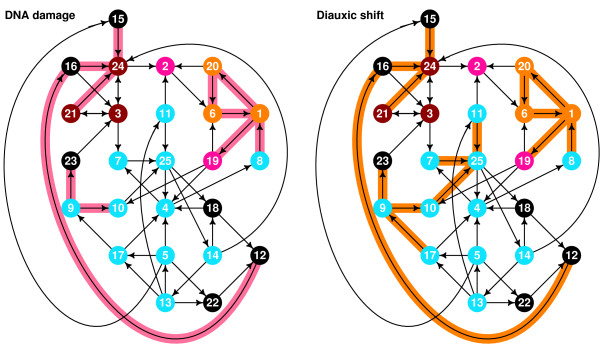
**Parts of LSCC that are active during exogenous condition (or, conditions with the fewest active cycles)**. DNA damage: The activity is, with small exception, subset of stress response, but without the cycle-closing activity of node 3. Diauxic shift: Part of stress activated too, like for DNA damage.

In Fig. 7 and Fig. 8 we can see the interactions of LSCC that are active in various physiological conditions. We can observe large difference between the subnetworks, both in the composition and in topological characteristics like average path length.

Luscombe *et al*. [6] measured the following topological characteristic in the subnetworks: the average length of shortest paths from TFs to TTs. By its very nature, LSCC is disproportionally involved in that characteristic. In the full network there are 113,000 such paths, and the average length is 4.81; among those paths, 100,910 go through LSCC, and their average length is 5.11, while only 12,090 paths does not go through LSCC and their average length is 2.63.

This domination of LSCC directly follows from the fact that every TF in LSCC and In-LSCC has a path to every TT that can be reached from LSCC, as a result, on the average one can reach 2968 TTs from these TFs through LSCC (in LSCC this number is contant, but in In-LSCC it can be smaller because some TTs reachable through LSCC may have shorter paths directly from In-LSCC). The average number of TTs reachable not through LSCC is 103 (for 117 TFs outside LSCC).

In other words, only 12% of shortest connections between TFs and TTs does not go through LSCC, and these paths contribute only 5.8% to the sum of lengths.

Because so many TF-to-TT paths go through LSCC, the differences between average path lengths that were observed for different subnetworks by Luscombe *et al*. [6] are largely caused by the different presence of these networks in the LSCC. In Table 5 we use PERCENTPATH to denote the percentage of the shortest paths from transcription factors to the terminal targets that either originate or go through LSCC, and PERCENTLENGTH to denote the similar percentage for the sum of lengths of shortest paths.

**Table 5:** Importance of LSCC in the paths of different subnetworks

subnetwork	**cc**	**sp**	**sr**	**ds**	**dd**
average path length	4.64	3.55	2.31	2.10	1.94
PERCENTPATH	87.1	69.4	72.1	57.8	54.6
PERCENTLENGTH	94.2	78.0	81.6	64.4	59.0

In each physiological subnetwork we consider the set *P *of pairs of the form TF-TT such that there is a chain of interactions (a path) from the TF of the pair to the TT; for *p *∈ *P *we define ℓ_*p*_, the length of the shortest path for this pair; moreover, *Q *is the subset of *P *such that the respective path has to go through LSCC. Then average path length = ∑_*p*∈*P*_ℓ_*p*_/|*P*|, PERCENTPATH = |*Q*|/|*P*|, and PERCENTPATH = ∑_*p*∈*Q*_ℓ_*p*_/∑_*p*∈*P*_ℓ_*p*_.

Table 5 shows that even in DNA damage and diauxic shift subnetworks the majority of shortest paths between TFs and TTs goes through LSCC; we may say that LSCC has a role of a *switchboard* (each node is TF in Table 6).

**Table 6:** Proteins that form nodes in Fig. 3

node	code	TF	node	code	TF
1	YBR049C	REB1	14	YLR183C	TOS4
2	YDR207C	UME6	15	YLR256W	HAP1
3	YDR259C	YAP6	16	YML007W	YAP1
4	YDR501W	PLM2	17	YML027W	YOX1
5	YER111C	SWI4	18	YNL068C	FKH2
6	YGL073W	HSF1	19	YNL216W	RAP1
7	YIL122W	POG1	20	YOL004W	SIN3
8	YJR060W	CBF1	21	YOR028C	CIN5
9	YKL043W	PHD1	22	YOR372C	NDD1
10	YKL062W	MSN4	23	YPL177C	CUP9
11	YKL112W	ABF1	24	YPR065W	ROX1
12	YLR131C	ACE2	25	YPR104C	FHL1
13	YLR182W	SWI6			

## Conclusion

We inspected graph-theoretic properties of the cycles in the transcription network of *Saccharomyces cerevisiae*. While in general cycles are "avoided" by the network, interactions common to all phases of the cell cycle form a big exception, and interactions specific to the stress response form a smaller exception. In spite of their modest number (they involve 25 of 142 transcription factors that were included in the data set), the transcription factors that are included in cycles have a large topological impact: most of the shortest paths between transcription factors and terminal targets go through them.

One should compile many kinds of data to establish the exact role of the cycles of transcription interactions in controlling life processes. In particular, cell cycle, which is closely related to cancer, possesses a long cycle that can be easily interrupted at many different points, and the process itself can be interrupted by a number of different conditions (like DNA damage).

We have shown that LSCC is a key part of the regulatory network and that it can be divided into functional subunits. Further work will yield fuller and clearer picture of these subunits and their interactions under various conditions.

## Methods

### Data

We used supplementary materials for [6] ; we also used supplementary materials of [7,15] and the list of yeast homologs of human cancer genes personally communicated by Haiyuan Yu.

### Graph-theoretic definitions

A *graph *of a network consists of *nodes *(which correspond to TFs, transcription factors and TTs, terminal targets) and directed edges/interactions.

A *path *in a graph is a sequence of nodes (*u*_0_, ..., *u*_*k*-1_) such that each consecutive pair (*u*_*i*-1_, *u*_*i*_) is an edge. If additionally there exists an edge (*u*_*k*-1_, *u*_0_) we say that this is a *cycle*.

A single node (*u*) forms a *degenerate *cycle.

Nodes in a graph are partitioned into *strongly connected components*, or SCC's. A node *u *is contained in SCC(*u*) which is the union of the node sets of all cycles that contain *u*.

SCC's with one node are called trivial.

For graph *G *we define strong component graph *G*_SCC_, the graph of SCC's of *G*. Nodes of *G*_SCC _are scc's of *G*, and edges are pairs of the form (SCC(*u*), SCC(*v*)) such that (*u, v*) is an edge of *G*.

*G*_SCC _cannot have cycles of its own, and therefore it is easy to compute longest paths in that graphs (the algorithm is considered folklore). The paths lengths in that graph are used in Fig. 1.

We use LSCC to denote the largest strongly connected component in a graph. We apply this definition when the majority of elements of non-trivial scc's belongs to one of them, so there is no ambiguity as to which one is "the largest".

### Algorithms

To compute non-trivial scc's we first obtained a "dictionary" protein code ↔ number followed by pairs of numbers representing the edges. We computed scc's and the graph of scc's using the method described in section 22.5 of Cormen *et al*. [16].

Shortest paths used in subsection on Position of LSCC in the hierarchy were computed using breadth first search.

### Defining motifs, generating random graphs

We define a feed-forward loop (3 for short) as a triple of nodes {*u*_0_, *u*_1_, *u*_2_} such that there exists three edges: two form a path (*u*_0_, *u*_1_, *u*_2_) while the third forms a shortcut, (*u*_0_, *u*_2_). A bi-fan is a quadruple of nodes (*u*_0_, *u*_1_, *v*_0_, *v*_1_) such that all of the 4 possible edges of the form (*u*_*i*_, *v*_*j*_) exist.

When we count ffl's and bi-fans we remove the self-loops (edges of the form (*u, u*)) from the graph.

Moreover, every triple/quadruple is counted separately, even when they share nodes.

To count ffl's and bi-fans we made a table Overlap that for a pair of TFs stored the number of common targets. For every positive entry *k *= *Overlap*(*a, b*) we add *k*(*k *- 1)*/*2 to the count of bi-fans, and if there is an edge from *a *to *b*, we add *Overlap*(*a, b*) to the count of ffl's.

We generated networks to make statistic comparisons. First, we generated random networks, or R. For Luscombe network, we permutated TF entries of adjacency lists at random. After permutation, lists could contain errors; a TF that "owns" the respective list, or a TF that has another copy earlier on the list. We repeated random permutations until error-free list were obtained, a process that took 1–2 seconds.

For Yu and Balaji, this provably unbiased approach [11] had no results within 30 minutes, so we used a variation of metropolis random walk. Starting from the original network, we repeatedly selected pairs of edges at random to swap their endpoints; a swap introducing new errors was performed with probability *β *and rejected otherwise. We set *β *so the process would result in an error-free network in a reasonable time (several seconds or several millions attempts on the average)

Random networks were modified to boost the number of motifs, either feed-forward loops (version F) or bi-fans (version B). Boosting was performed via a metropolis process in which a randomly selected swap was rejected if it decreased the number of desired motifs by *k *(more precisely, such a swap was rejected with probability 1 - *α*^*k *^for some *α*), or if it increased the number of errors by *l *(a swap was rejected with probability 1 - *β*^*l*^). Parameter *α *was adapted by the algorithm; decreased if the number of motifs was too small and not growing, and increased when it was too large.

## Abbreviations

TF, transcription factor. TT, terminal target. LSCC, large(st) strongly connected component. SCC, strongly connected component. SSCC, small cyclic SCC's. Various networks in Luscombe *et al*. [6] data: **al**, all interactions, **cc**, interactions of the cell cycle, **dd**, interactions of the DNA damage, **ds**, interactions of the diauxic shift, **sp**, interactions of sporulation, **sr**, interactions of the stress response. Ccc, interactions in common in 5 stages of the cell cycle. ITF, interactions from TF to TF. For a class of interactions *X *(like ITF and LSCC) and a subnetwork **yy **(like **cc **and **dd**), *X*_**yy **_denotes the intersection. *F*, feedback node set. MPL, the maximal path length for a path that includes a given TF. IN-LSCC, the in-component of LSCC. OUT-LSCC, the out-component of LSCC. SIMPLE, TFs whose longest paths to which they belong is at most 1 or 2. INT, TFs whose longest paths to which they belong is at most 3. EXCP, TFs that are not in any of IN-LSCC, LSCC, OUT-LSCC, or SIMPLE. ffl, feed-forward loop. In tables, we used Luscombe, Yu and Balaji to refer to networks from the data sets published in [6,7,15] respectively, and we used R, F and B to refer to random models generated with simple metropolis method (R), a variation of that method that increased the number of ffls (F) to the actually observed value, and a similar variation for the bi-fan motifs (B). The terms PERCENTPATH and PERCENTLENGTH are explained in detail in the caption of Table 5.

## Authors' contributions

JJ participated in the design of the study, analyzed the network, and drafted the manuscript. PB participated in the design of the study and the statistical analysis, and coordinated the project. All authors read and approved the final manuscript.
